# Testing an emerging animal model for use in the allergenicity assessment of food

**DOI:** 10.1186/1710-1492-7-S2-A1

**Published:** 2011-11-14

**Authors:** David E Lefebvre, Nikia Ross, Laurie Coady, Cheryl Armstrong, Susan Gurofsky, Ivan Curran, Tim Schrader, Don Caldwell, Genevieve S Bondy

**Affiliations:** 1 Toxicology Research Division, Bureau of Chemical Safety, Food Directorate, Health Canada, Ottawa, ON, K1A 0L2, Canada; 2 Scientific Services Division, Bureau of Chemical Safety, Food Directorate, Health Canada, Ottawa, ON, K1A 0L2, Canada

## Background

The regulatory assessment of novel food includes tests for allergy. The World Health Organization suggests tests in an animal model of allergy despite the lack of a validated model. We aimed to confirm if C3H/HeJ mice would respond to food of high allergenic potential (peanut), but not to food of low allergenic potential (turkey, potato, spinach).

## Methods

In the first study, C3H/HeJ mice were orally treated, once per week for two weeks, with adjuvant and 0 or 2 mg of peanut or turkey. A second study used adjuvant and 0, 0.1, 1 or 2 mg of peanut, potato or spinach. Blood IgE antibodies and spleen interleukin-4 were quantified.

## Results

Mice treated with 2 mg peanut developed peanut-specific IgE levels which were significantly higher than control mice (p<0.001, n=10/group). Mice treated with 2 mg turkey developed a similar IgE response to turkey (p<0.001, n=10/group). In the second study, allergy was only triggered in one of ten mice treated with 2 mg peanut. Two of ten mice exposed to 1 mg potato had a response. There were no IgE responders to spinach. Spleen cells from both the peanut- and the spinach-treated mice secreted more allergy-promoting interleukin-4 than controls (p<0.01, n=7-24/group). Levels were not modified in potato-treated mice.

## Conclusions

C3H/HeJ mice developed food allergy markers to peanut. However, the incidence varied between experiments. Some mice developed a similar response to foods with low allergenic potential. Thus, this model may not be appropriate for safety assessment of novel food.

